# Prescribing differences among older adults with differing health cover and socioeconomic status: a cohort study

**DOI:** 10.1186/s12877-023-04441-9

**Published:** 2023-11-17

**Authors:** Ciaran Prendergast, Michelle Flood, Logan T. Murry, Barbara Clyne, Tom Fahey, Frank Moriarty

**Affiliations:** 1 School of Pharmacy and Biomolecular Sciences, RCSI University of Medicine and Health Sciences, 123 St Stephen’s Green, Dublin, Ireland; 2 Department of General Practice, RCSI University of Medicine and Health Sciences, 123 St Stephen’s Green, Dublin 2, Ireland

**Keywords:** Polypharmacy, Medications, Prescribing, Primary care, General practice, Older adults

## Abstract

**Introduction:**

As health reforms move Ireland from a mixed public-private system toward universal healthcare, it is important to understand variations in prescribing practice for patients with differing health cover and socioeconomic status. This study aims to determine how prescribing patterns for patients aged ≥ 65 years in primary care in Ireland differ between patients with public and private health cover.

**Methods:**

This was an observational study using anonymised data collected as part of a larger study from 44 general practices in Ireland (2011–2018). Data were extracted from electronic records relating to demographics and prescribing for patients aged ≥ 65 years. The cohort was divided between those with public health cover (via the General Medical Services (GMS) scheme) and those without. Standardised rates of prescribing were calculated for pre-specified drug classes. We also analysed the number of medications, polypharmacy, and trends over time between groups, using multilevel linear regression adjusting for age and sex, and hospitalisations.

**Results:**

Overall, 42,456 individuals were included (56% female). Most were covered by the GMS scheme (62%, n = 26,490). The rate of prescribing in all drug classes was higher for GMS patients compared to non-GMS patients, with the greatest difference in benzodiazepine anxiolytics. The mean number of unique medications prescribed to GMS patients was 10.9 (SD 5.9), and 8.1 (SD 5.8) for non-GMS patients. The number of unique medications prescribed to both GMS and non-GMS cohorts increased over time. The increase was steeper in the GMS group where the mean number of medications prescribed increased by 0.67 medications/year. The rate of increase was 0.13 (95%CI 0.13, 0.14) medications/year lower for non-GMS patients, a statistically significant difference.

**Conclusion:**

Our study found a significantly larger number of medications were prescribed to patients with public health cover, compared to those without. Increasing medication burden and polypharmacy among older adults may be accelerated for those of lower socioeconomic status. These findings may inform planning for moves towards universal health care, and this would provide an opportunity to evaluate the effect of expanding entitlement on prescribing and medications use.

**Supplementary Information:**

The online version contains supplementary material available at 10.1186/s12877-023-04441-9.

## Introduction

With changing population profiles and increasingly costly medical interventions, high and middle-income countries are facing challenges in providing affordable healthcare to their populations [1]. By 2041, citizens over 65 years of age will make up 22% the Irish population; a doubling of the 2006 figure [2]. Delivering functional and affordable systems of universal healthcare requires identifying the optimal healthcare system which balances patients’ needs with services and costs covered [3]. In Ireland, political discussions surrounding healthcare reform culminated in the *Sláintecare* report in 2017, which provided a roadmap to a future single-payer system of universal healthcare, based on need and not on ability to pay [4].

At present, the Irish healthcare system is two-tiered and incorporates a mix of both public and private elements [5]. Notably, access to prescription medications varies considerably for individuals based on income and age. Some patients with full public health cover pay only a small prescription charge for each medication. From 2012, single people with a weekly income below €184 (age < 66 years), €201.50 (age 66–69 years) or €500 (age ≥ 70 years) were eligible, while for couples, weekly income thresholds were €266.60, €298, and €900 for those aged < 66 years, 66–69 years, and ≥ 70 years respectively. Alternatively, individuals who do not meet these income and age criteria pay out-of-pocket for the cost of their prescription medications, up to a monthly household cap. Differences in prescription medication use between these groups may arise due to differing individual characteristics (i.e. socioeconomic status), but also the effect of differing healthcare.

Existing literature has identified variation in medication prescribing for individuals with public and private health cover and access. Previous studies in countries in Africa and Sweden found physicians working in the private sector are less likely to adhere to guidelines, while also being less likely to prescribe rationally for certain conditions [6, 7]. In Ireland, polypharmacy and potentially inappropriate prescribing have increased in recent years; however, the evidence for prescribing variation between the public and private sector is mixed [8]. A 2008 study found no evidence of a difference in prescribing rates, but higher inappropriate prescribing of antipsychotics to individuals in private residential care settings compare to public [9]. A more recent study identified that public patients in Ireland had a 21–38% greater risk of polypharmacy compared to patients with private health cover. The study authors concluded that public health cover in Ireland led to greater medication use in people aged 50–69 years [10]. International evidence has also examined prescription practices, and in several Swedish studies, private providers were found to prescribe a higher number of medications, though less cost-effectively, than public general practitioners (GPs) [11, 12].

The majority of studies comparing prescriptions in the public and private sectors have been carried out in low- or middle-income countries, where a series of comprehensive meta-analyses support the idea that there is measurable variability in prescribing practice between sectors [13, 14]. The Irish system presents a unique opportunity to evaluate prescribing differences among patients with differing healthcare entitlements, cared for by the same providers. An understanding of differences in prescribing patterns between public and private patients in Irish general practice is important if future health reform extends coverage of prescription medications entitlement.

## Aim and objectives

This study aims to determine how prescribing practices for patients aged 65 years and over in primary care in Ireland differ between patients with public and private health cover.

The objectives are to assess differences in the:

- Rate of prescribing of common drug classes.
- Prevalence of individual drugs within each common drug class of interest.
- Number of medications prescribed.

## Methods

### Study design, population, and setting

This was an observational study reported in line with the STrengthening the Reporting of OBservational studies in Epidemiology (STROBE) statement [15]. Anonymised data were collected as part of a larger study from 44 general practices in the Republic of Ireland using the patient management software Socrates (www.socrates.ie) between January 2011 and April 2018. Ethical approval was obtained from the Irish College of General Practitioners Research Ethics Committee. Participating practices from the catchment areas of Dublin (n = 30), Galway (n = 11), and Cork (n = 3) hospitals represented 91% of those contacted. Ireland has a mixed public-private health system, and a proportion of the population are entitled to public health cover, with eligibility based on household income and age. The General Medical Service (GMS) scheme covers the most socioeconomically deprived people, approximately one third of the population, and entitles them to GP visits and a range of health services free at the point of access, and prescription medications (with a small co-payment of €2.50) [16]. The Doctor Visit Card (DVC) scheme covers people with higher, but still limited, means, who are entitled to free GP visits but pay for other health services and their medications. All other individuals pay for healthcare and prescription medications (with a household cap of €144 per month applying during the study period). Ireland’s state drug schemes stipulate that no more than one month’s supply can be covered in any month, and this requirement means there is no incentive for those with different health cover to receive prescriptions written differently (e.g. a larger quantity with a smaller number of issues), as pharmacies will still dispense on the same basis.

Data were extracted from the patient management system relating to demographics, consultations, prescribing and hospitalisations for patients aged 65 years and older. Patients were included in the present analysis if they had prescriptions issued on at least two dates during the study period, and had demographics (age and sex) and date of prescribing data recorded. Observations with a date of prescription outside of the study period were removed from the analysis.

### Study variables

Prescription records in the dataset are at the medication level and included date of prescription, number of issues (i.e., how many times a prescription could be dispensed), product name, and generic name. Medications were coded using the Anatomical Therapeutic Chemical (ATC) classification, a system developed by the World Health Organisation for drug utilisation research and monitoring. ATC codes are organised by physiological system and are hierarchical, with the full seven-character ATC code (fifth level) identifying the active substance, and the five-character ATC code (fourth level) identifying the chemical subgroup level (usually equivalent to the drug class). Age (grouped as 65–69, 70–74, 75–79, 80–84, 85–89 and 90 years and over) and sex were extracted as demographic variables from the GP records, as was the type of health cover a patient had: GMS scheme (considered “public”), DVC scheme, or neither of these (considered to be “private”). We grouped the DVC scheme cohort with the private cohort as a “non-GMS” category, as although GP visits are covered by the state, medications are not in this instance. We also created a time-varying variable, counting the number of hospitalisations each individual had during the study period.

We calculated the rate of prescribing for drug classes at the fourth-level ATC code, both overall and separately for GMS and non-GMS patients. We pre-specified 12 drug classes of interest before commencing the study (Table 1), based on their high prevalence of use, their inclusion in Ireland’s Preferred Drugs Initiative (Health Service Executive Medicines Management Programme), [17] or potential for sub-optimal prescribing.

We calculated the number of unique drug classes (at the fourth-level ATC code) each patient had been prescribed over the previous 12 months on a rolling basis across the study period, which was used as the number of medications each patient was prescribed. The rationale for this definition was to avoid inflation of the number of medications over the 12-month time window by therapeutic switching within a drug class, consistent with our previous work [8]. The number of medications prescribed was also converted into a categorical variable with prescription of 5–9 medications being classed as ‘polypharmacy’ and 10 or more medications being classed as ‘major polypharmacy’.

**Table 1:** Pre-specified drug classes of interest and corresponding ATC codes

ATC code	Drug class
C10AA	Statins
A02BC	Proton pump inhibitors
C07AB	Beta blocking agents, selective
B01AA, B01AE, B01AF	Direct oral anticoagulants
C09AA and C09B	ACE inhibitors (both single agent products and combinations)
C08CA	Dihydropyridine calcium channel blockers
N05CF	Z-drug hypnotics
N06AB	Selective serotonin reuptake inhibitors
N05BA	Benzodiazepine anxiolytics
C09CA and C09D	Angiotensin receptor blockers (both single agent products and combinations)
R03AC and R03AK	Adrenergics in combination with corticosteroids or other drugs, and/or anticholinergics
J01	Antibacterials for systemic use

### Statistical analysis

First, we described patient characteristics, both overall and separately for GMS and non-GMS patients. For objective 1, we directly standardised rates of prescribing (based on number of prescriptions and number of repeats/issues per prescription) for drug classes among GMS patients to the non-GMS population, using age group, sex, and calendar year, generating 95% confidence intervals (95% CIs) for the rates in both groups. Including year as a standardisation variable accounted for the amount of time patients were present in the dataset. The ratio of the prescribing rate for each drug class among the GMS versus non-GMS patients was plotted as a bubble graph. The same analysis was carried out comparing the GMS group to the DVC group alone, and the private group alone.

For objective 2, we determined the prevalence of individual medications (fifth-level ATC codes) within each drug class of interest, and assessed any difference between health cover groups in the distribution of prescribing within drug classes using a chi-squared test. A single practice, which was missing number of repeats/issues data, was excluded from this drug class analysis.

For objective 3, we used monthly values for the number of unique drug classes (based on the fourth-level ATC code) each patient had been prescribed over the previous 12 months to plot the mean number over time for GMS and non-GMS patients. We also plotted the proportion of GMS and non-GMS patients with polypharmacy over time in categories of 1–4, 5–9, 10–14 and 15 + medications. We also summarised the mean number of medications prescribed per person over the full study period for the GMS and non-GMS groups, taking a mean of the number of medications each time a prescription was issued (excluding observations in the 12 months after the first date of prescription for an individual, where a full 12-month period for calculating number of medications was not yet available).

Lastly, we used a multilevel linear regression analyses to assess whether the number of medications differed by health cover and over time. Data was hierarchical with monthly time points, nested within individual patients, nested within GP practices. The fixed covariates included date of prescription (scaled to 1 unit per year and continuous), health cover type (categorical, GMS and non-GMS), age (continuous in years) and sex (categorical, male and female). Random intercepts were included for the patient and practice level, and variance and variance partition coefficients were estimated for each level. A second model was also fitted to include an interaction between date of prescription and health cover, assessing whether any change in number of medications prescribed over time differed according to health cover. A third model included a hospitalisations variable, to examine how this may explain differences in the number of medications between health cover groups. When modelling, the mean number of unique medications prescribed to individuals over time, observations occurring less than 12 months after the first for an individual were removed as incomplete 12-month periods. Analyses were conducted using the lme4 package in R, [18, 19] and statistical significance was assumed at p < 0.05.

## Results

The analyses included data on 42,456 individuals, of which 44% (n = 18,695) were male and 56% (n = 23,761) were female. The majority (62%, n = 26,490) of individuals were covered by the GMS scheme, while the remaining 15,966 were non-GMS (70% private and 30% DVC). The mean age of the GMS cohort was 78.9 years (SD 8.1) and the mean age of the non-GMS cohort was 79.4 (SD 9.2). There was a higher proportion of females in the GMS group (57%) compared to the non-GMS group (52.7%). Demographics and health cover status for participants are included in Table 2.

**Table 2:** Descriptive characteristics of included participants

Characteristic	Total (n = 42,456)	GMS (n = 26,490)	Non-GMS (n = 15,966)
Age (years), mean (SD) [95%CI]	79.0 (8.3) [78.9–79.1]	78.9 (8.1) [78.8–79.0]	79.4 (9.2) [79.3–79.5]
Age group, n (%, 95% CI))			
65–69 years	7,965 (18.8%, 18.4–19.1%)	3,591 (13.3%, 12.9–13.7%)	4,374 (27.4%, 26.7–28.1%)
70–74 years	9,070 (21.4%, 21.0-21.8%)	5,232 (19.4%, 19.0-19.9%)	3,838 (24.0%, 23.4–24.7%)
75–80 years	7,729 (18.2%, 17.8–18.6%)	5,328 (19.7%, 19.3–20.3%)	2,401 (15.0%, 14.5–15.6%)
80–84 years	6,919 (16.3%, 15.9–16.7%)	5,057 (18.8%, 18.3–19.2%)	1,862 (11.7%, 11.2–12.2%)
85–89 years	5,480 (12.9%, 12.6–13.2%)	3,916 (14.5%, 14.1–15.0%)	1,564 (9.8%, 9.3–10.3%)
90 + years	5,294 (12.5%, 12.2–12.8%)	3,366 (12.5%, 12.1–12.9%)	1,928 (12.1, 11.6–12.6%)
Female, n (%, 95% CI)	23,761 (56.0%, 55.5–56.4%)	15,353 (57.0%, 56.4–57.6%)	8,408 (52.7%, 51.9–53.4%)
Male, n (%, 95% CI)	18,695 (44.0%, 43.6–44.5%)	11,587 (43.0%, 42.4–43.6%)	7,558 (47.3%, 46.6–48.1%)
Health cover, n (%, 95% CI)			
General Medical Services scheme	26,490 (62.4%, 61.9–62.9%)	26,490 (100.0%)	0
Doctor Visit Card	4,743 (11.2%, 10.9–11.5%)	0	4,743 (29.7%, 29.0-30.4%)
Private	11,223 (26.4%, 26.0-26.9%)	0	11,223 (70.3%, 69.6, 71.0%)

### Drug class prescribing

**Fig. 1:**
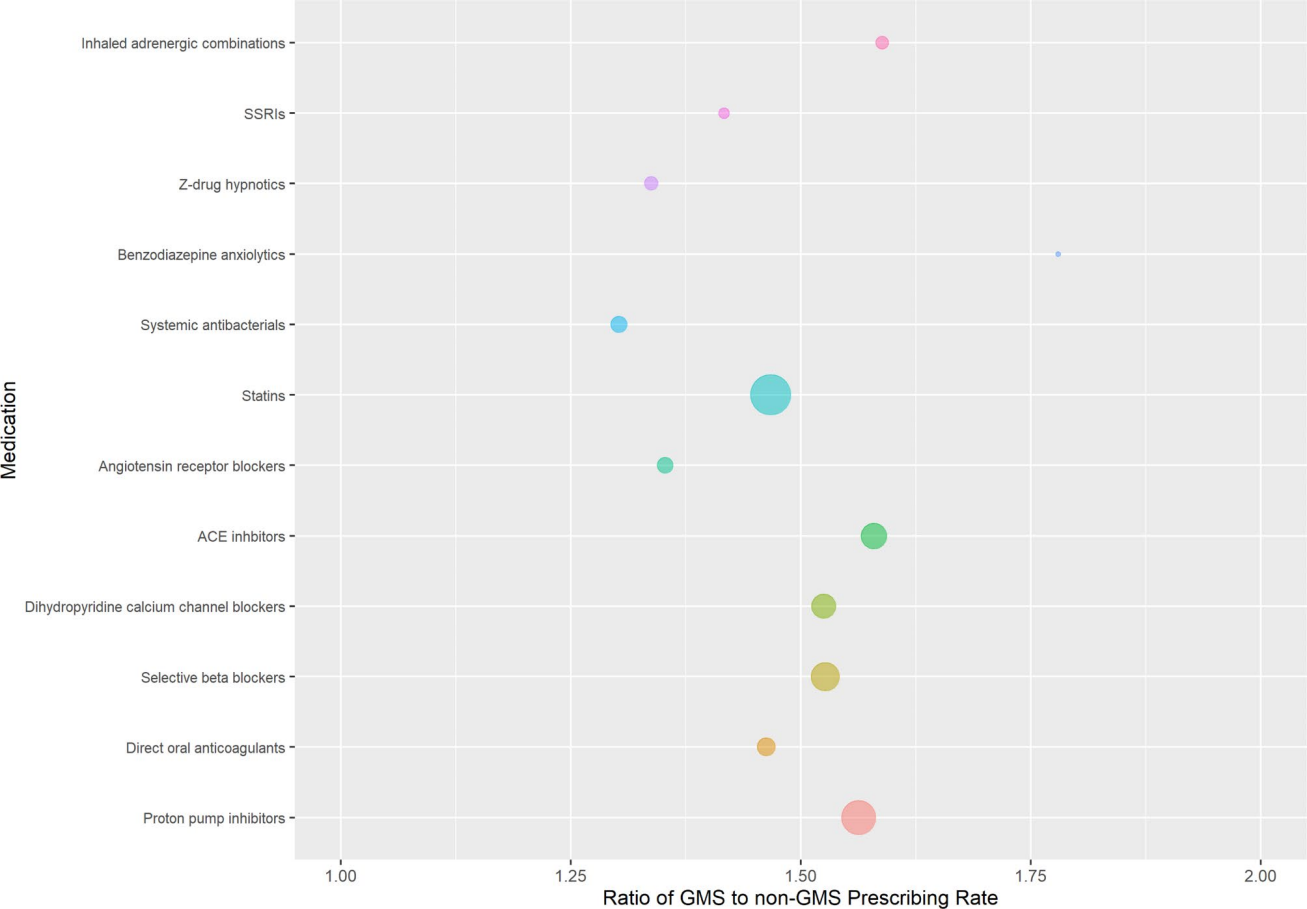
Ratio of General Medical Services (GMS) to non-GMS prescribing rates for pre-specified drug classes, with bubble size indicating the rate of prescribing of each class among GMS patients

The rate of prescribing in all pre-specified drug classes was higher for GMS patients compared to non-GMS patients. Figure 1 shows the ratios of GMS to non-GMS prescribing rates for these classes. In all cases, the rate of prescribing was at least 1.3 times higher in the GMS group, with the smallest difference in systemic antibacterials. We saw the greatest disparity in benzodiazepine anxiolytics where the rate of GMS prescribing was 1.78 times higher; a rate of 996 per 1000 person-years in the GMS group versus a rate of 559 per 1000 person-years in the non-GMS group. The next largest difference was inhaled adrenergic medications combined with corticosteroids and/or anticholinergics, with a rate 1.58 times higher in the GMS group. Number of prescriptions and standardised rates for each drug class in each group are reported in supplementary Table 1. In sensitivity analysis, ratios of GMS to DVC rates were higher than the corresponding ratio of GMS to private rates in most cases, with the exception of statins, angiotensin receptor blockers, and dihydropyridine calcium channel blockers (Supplementary Fig. 1). A further sensitivity analysis considering prevalence (i.e. number of people prescribed the drug class, rather than the rate of prescribing per 1,000 person-years) again showed higher prevalence in the GMS group versus non-GMS across all drug classes. The difference were more modest, ranging from 1.04 to 1.30, the largest difference being in inhaled adrenergic combinations (Supplementary Fig. 2 and Supplementary Table 2).

**Fig. 2:**
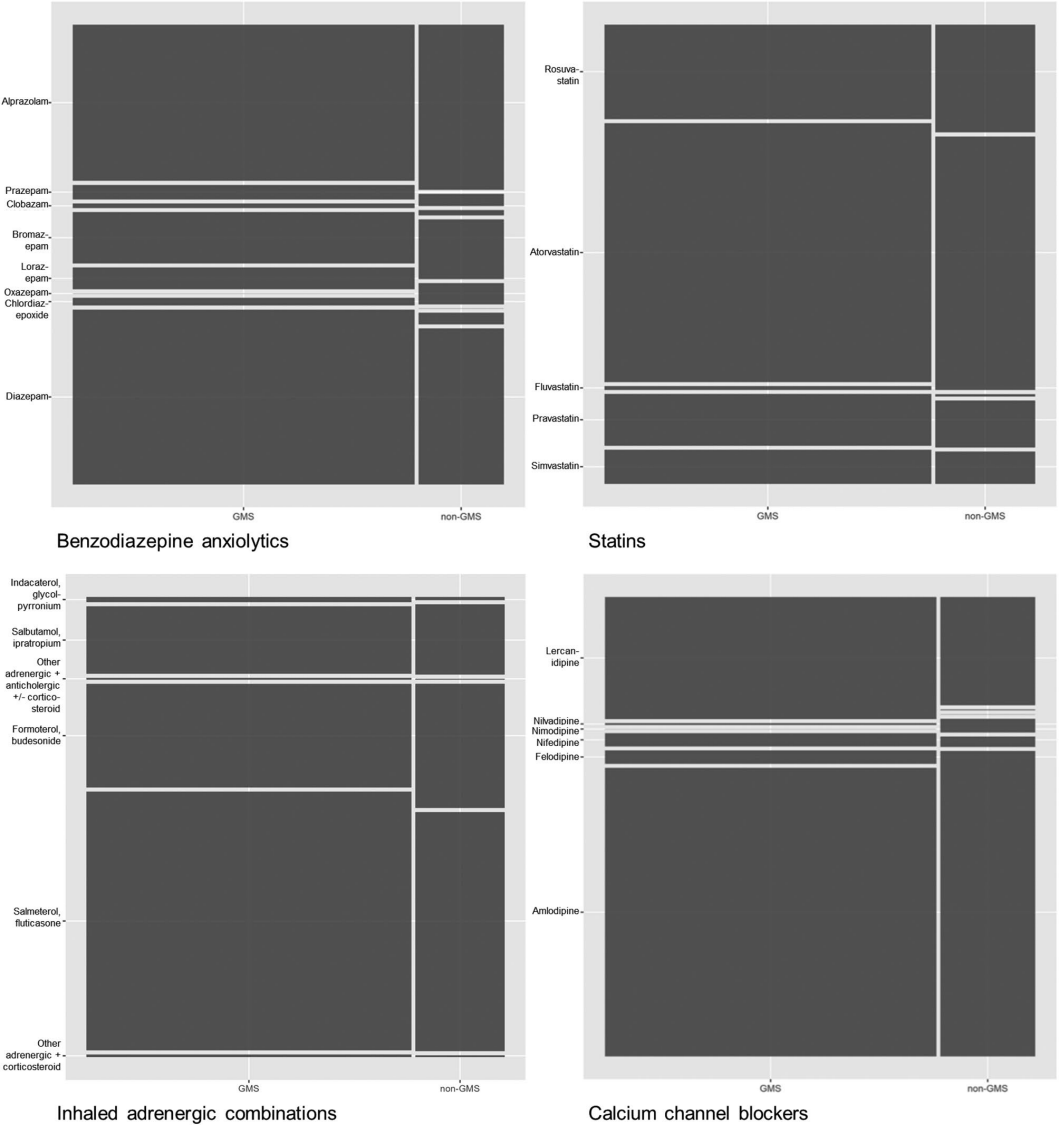
The relative proportions of individual medication prescribing (indicated by fifth-level ATC codes) for (clockwise from top left) benzodiazepine anxiolytics, statins, dihydropyridine calcium channel blockers, and inhaled adrenergic combinations in the General Medical Services (GMS) and non-GMS groups

As examples, the mosaic plots below (Fig. 2) show the relative proportions of medications (fifth-level ATC codes) that make up four of the pre-specified drug classes (benzodiazepine anxiolytics, statins, inhaled adrenergic combinations, and calcium channel blockers). For benzodiazepine anxiolytics, diazepam made up a significantly greater proportion of prescribing in the GMS group compared to the non-GMS group (a difference of 5% points), whereas the reverse was true of alprazolam (which was 2% points higher among non-GMS patients). Within calcium channel blockers, amlodipine made up a significantly greater proportion of prescribing within the non-GMS cohort (a difference of 4% points). For inhaled adrenergic combinations, salmeterol/fluticasone made up significantly more prescribing in the GMS group (4.5% points higher), whereas formoterol/budesonide made up significantly more of non-GMS group prescribing for this drug class (5% points higher). For statins, the largest difference was rosuvastatin accounting for 3% points more of statin prescribing in the non-GMS group. Mosaic plots for the other drug classes are included as supplementary Fig. 3, and frequency tables for medications within each drug class by health cover are included as supplementary Table 3.

## Number of medications

**Fig. 3:**
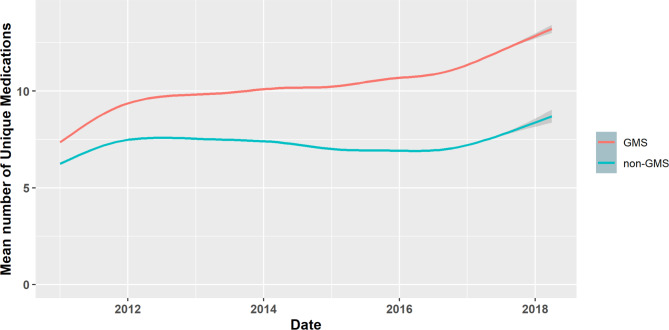
Time trend comparing the changes in number of unique medications prescribed to both General Medical Services (GMS) and non-GMS groups over time, with grey shading indicated 95% confidence intervals

The number of unique medications prescribed to both the GMS and non-GMS cohorts increased over time, as depicted by the time trend in Fig. 3. The increase was more pronounced and more sustained in the GMS group, rising from a mean of 7.3 (SD 5.8) medications in January 2011 to a level of 14.2 (SD 7.1) in April 2018 compared to the non-GMS group rising from 5.8 (SD 4.8) to 9.2 (SD 6.6). Fig. 3, shows the fitted line for the number of medications over time for each group.

**Fig. 4:**
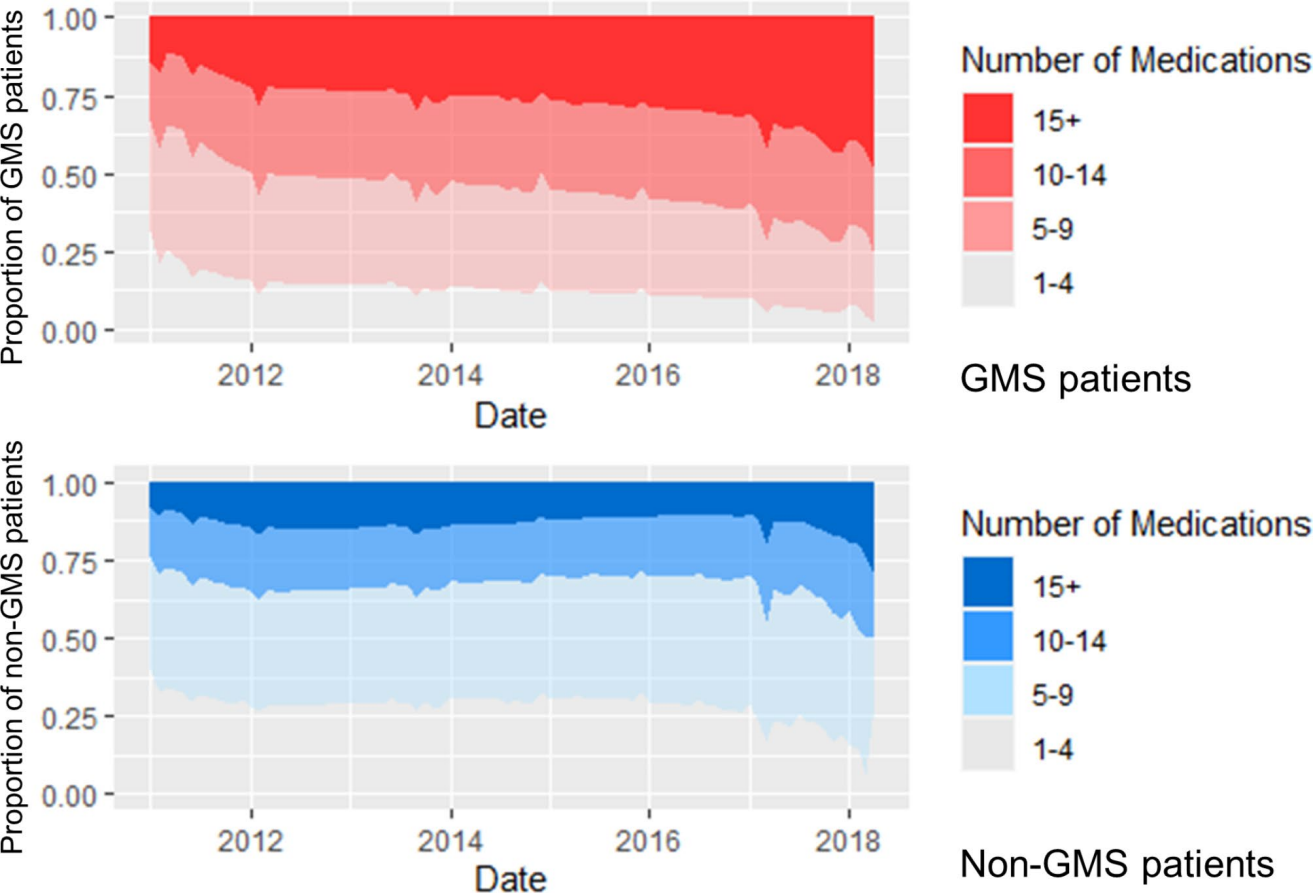
Proportion of patients with levels of polypharmacy over the study period for General Medical Services (GMS) (top) and non-GMS groups (bottom)

The rates of polypharmacy (≥ 5 medications from different fourth-level ATC drug groups), and major polypharmacy (≥ 10 medications from different fourth-level ATC drug groups) over time are shown in Fig. 4. The GMS group began the study period with higher rates of major polypharmacy and this became more pronounced over time. The rate of major polypharmacy in the GMS group increased from 33.2% in January 2011 to 76.5% in April 2018.

**Fig. 5:**
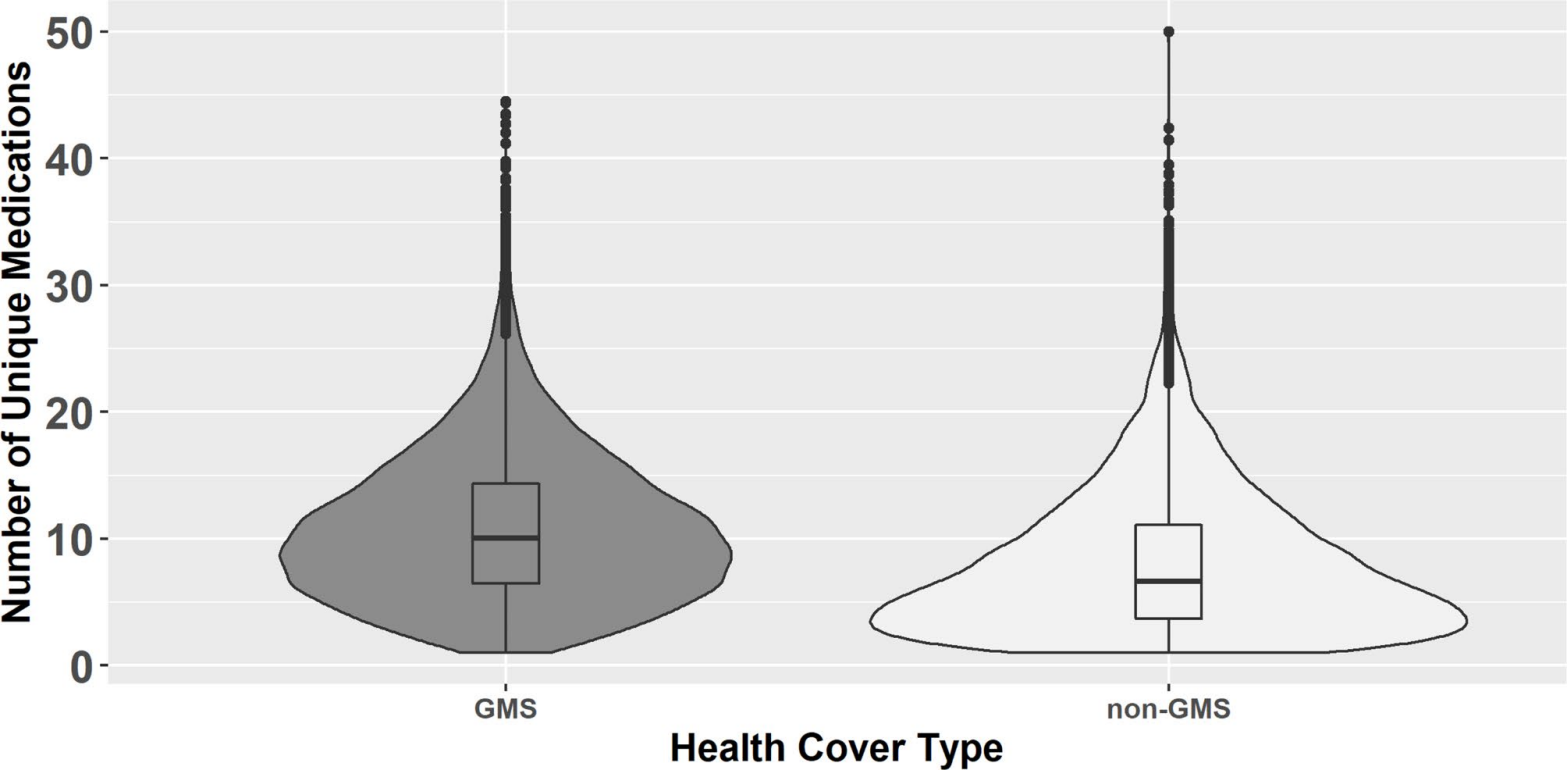
Violin plot showing the number of unique medications prescribed to General Medical Services (GMS) and non-GMS patients

The mean number of medications (from unique drug classes) prescribed to GMS patients over the full study period was 10.9 (SD 5.9), compared to a mean of 8.1 (SD 5.8) among non-GMS patients. Similarly, the median number of unique drug classes prescribed (Fig. 5) was higher among GMS patients at 10.1 (IQR 6.5 to 14.3) compared to non-GMS patients (median 6.6, IQR 3.7 to 11.1).

The results of the multilevel regression model are shown in Table 3. Based on variance partition coefficients in Model 1, 4% of variation was between practices, 75% was between patients within practices, and 21% was within patients over time. There was a statistically significant increase in number of unique medications over time (0.65 additional medications per year, 95% CI 0.64, 0.65), with non-GMS patients (compared to GMS patients) being prescribed 1.93 (95% CI 2.00, 1.87) fewer medications. Being female was associated with a higher number of medications (0.91 additional medications, 95% CI 0.85, 0.96) compared to males. In model 2, including an interaction term between time and health cover, the VPC were similar to model 1. In this model, mean number of medications prescribed increased by 0.67 medications/year for GMS patients. The rate of increase was 0.13 (95%CI 0.13, 0.14) medications/year lower for non-GMS patients, a statistically significant difference. In model 3, including a variable counting the number of hospitalisations, the increase in medications over time and the difference in the rate of increase by health cover were both attenuated.

**Table 3:** Characteristics associated with number of unique medications over time in multilevel linear regression

	Change in number of medications (95% confidence interval)		
Covariates	Model 1	Model 2	Model 3
Time, per year	0.65 (0.64, 0.65)	0.67 (0.67, 0.67)	0.54 (0.54, 0.54)
Non-GMS	-1.93 (-2.00, -1.87)	-1.58 (-1.64, -1.51)	-1.58 (-1.65, -1.52)
Age	0.22 (0.22, 0.23)	0.22 (0.22, 0.22)	0.21 (0.21, 0.22)
Sex (female)	0.91 (0.85, 0.96)	0.90 (0.85, 0.95)	0.97 (0.92, 1.03)
Non-GMS:Time, per year, interaction		-0.13 (-0.14, -0.13)	-0.10 (-0.11, -0.10)
Hospitalisations			0.48 (0.47, 0.48)
**Variance (VPC)**			
Practice	1.5 (3.6%)	1.5 (3.5%)	1.4 (3.6%)
Patient	31.2 (75.4%)	31.1 (75.4%)	29.6 (74.9%)
Residual	8.7 (20.9%)	8.7 (21.0%)	8.5 (21.5%)

GMS, General Medical Services scheme; VPC, Variance Partition Coefficient

## Discussion

In this study, we found higher numbers of medications prescribed to older adults with public health cover (GMS) compared to those without. We also identified faster growth in the number of medications over time within the public GMS cohort. This is reflected in higher rates of prescribing of all of the pre-specified drug classes we examined, with the greatest difference in rates for inhaled adrenergic combination medications. Within drug classes, there were some differences in the percentage share of individual medications between health cover groups, however these did not consistently align with national preferred drug guidance. Similarly, this did not consistently align with less expensive options being used more by the public GMS cohort (who would not pay the full medication cost). The faster growth in medications over time for the public GMS group was partly explained by the higher rate of hospitalisation. We identified most variation in number of medications was between patients, rather than between practices or over time within patients, suggesting individual patient factors are explain more variation than differing prescriber habits.

Direct comparison with other research is challenging, as most examine prescribing differences between patients attending public versus private providers in other healthcare systems, rather than the same providers prescribing to those with differing healthcare entitlements, as in Ireland’s health system. Studies by Granlund (2009) and Hakansson et al. (2001) found a significantly larger number of unique medications were prescribed to public, rather than private patients. In the Irish setting, Mohan et al. (2021) also reported this between-group difference in number of medications and the faster growth in medications prescribed over time in the over 50s public cohort in Ireland [11, 12, 20].

One reason for the prescribing difference between health cover groups we identified may be that socioeconomic status is known to correlate with several measures of health, in Ireland [21]. As a result, the publicly covered population is likely to have a higher illness burden, requiring greater pharmaceutical intervention. This association is robust in the literature as made clear by Pathirana and Jackson, (2018), who performed a systemic review encompassing 24 cross-sectional studies, primarily in high-income settings, showing level of educational attainment and deprivation (as measures of socioeconomic status) were both associated with increased risk of multimorbidity [22]. Guthrie et al. (2015) identified an association between living in a deprived area and increasing polypharmacy among adults of all ages in a region of Scotland [23]. Also in Scotland, a study by Barnett et al. (2012) showed that the accumulation of chronic conditions was more substantial, and occurred earlier (by up to 15 years), in those of a lower socioeconomic status [24]. Given the overrepresentation of socioeconomic deprivation among those with public health cover in Ireland, this may partly explain higher rate of growth in medication burden among GMS patients.

Inhaled adrenergic combination medications showed the second largest or largest difference in prescribing (across prescribing rates or prevalence) of our chosen drug classes, which is striking, as there is a particularly strong negative correlation between socioeconomic status and respiratory diseases [25]. Previous evidence in Ireland has shown this relationship, and respiratory diseases as a whole are more common in Ireland than in many comparable developed nations in Europe [26]. By way of partial explanation, rates of smoking in Ireland have historically been shown to be significantly higher in those of lower socioeconomic status [27]. The smallest difference in prescribing rates was for systemic antibacterials, being 1.3-fold higher in GMS patients. Unlike most of the other drug classes examined, these are often short-term prescriptions (thus the impact of deprivation on illness burden may be amplified/propagated less). Further evidence from Scotland found an association between deprivation and rates of antimicrobial prescribing [28]. In contrast, a previous study in Ireland including individuals of all ages found private patients were more likely to receive an antibiotic prescription than GMS patients, however this was reversed among patients aged 65 years and over, consistent with our findings [29]. The less pronounced difference in prescribing rates may also be partly explained by the existence of primary care antimicrobial prescribing guidelines in Ireland since 2012 [30]. Although prescribing and cost guidance was also issued for inhaled medicines for chronic obstructive pulmonary disease during the study period, these more related to the choice of medication rather than the decision to prescribe or not, and so may not have impacted the large difference in inhaled adrenergic combinations.

An increase in medication burden post hospitalisation is a common occurrence [31, 32]. However, whether the increased medication burden is maintained after discharge is often not examined [33, 34]. We addressed this issue with a multilevel regression model that accounted for the association of hospitalisation with number of medications over time, and found a sustained positive effect. The appropriateness of the increased medication burden is unclear. Viktil et al., (2012), cite a similar number of medication changes upon discharge, commenting on a delay in receipt of discharge notes and speculate that failure to communicate between primary and secondary care contributes to potentially inappropriate prescribing. This finding is built upon by Coll et al., (2021), who show that the inclusion of instructions upon discharge accelerates the discontinuation of benzodiazepines and Z-drugs in older adults [35]. Further work by Perez et al., (2018), suggest that the risk of potentially inappropriate prescribing increases with rates of hospitalisation and degree of multimorbidity [36]. Patients were found to be 72% more likely to have been prescribed a potentially inappropriate medication after a single hospitalisation.

Although increased prescribing post hospitalisation could be due to short-term treatments relating to the admission, a study by Corsonello et al. (2007) identified that most new medications related to chronic conditions, and so the higher prescribed largely represented a ‘true and stable’ increase [32]. This may be reflected in our study, as the cohort that accrues chronic conditions earlier and to a greater degree, show the largest increase in polypharmacy. Our study did not account for changes to medication regimens that produced no overall change in medication burden, though this has been put forward as an indicator for identifying patients at risk of potentially inappropriate prescribing [37].

Our study provides a longitudinal analysis of polypharmacy, a view which is under reported in the literature. Falster et al., highlight that although the medications that make up patients’ polypharmacy change regularly over time, once reached, chronic polypharmacy is often permanent among older patients [38]. A limitation of our study is that we were unable to examine which factors relating to public healthcare entitlement (i.e. increased access to healthcare and medications, or the underlying differences in socioeconomic status) have the greatest relationship with prescribing differences. Therefore, it is not possible to conclude what prescribing rates would be if healthcare entitlement was widened. Although we found higher prescribing rates across our pre-specified drug classes, other classes could potentially show different patterns. However our overall findings for number of medications and polypharmacy support a widespread relationship between health cover status and medication burden. Our analysis was also limited to those aged 65 years and over, and therefore cannot be generalised to younger patients. However, the older age group account for the majority of medication utilisation. Lastly, our data related to prescribing rather than dispensing, as used in much of the literature, and so there is potential that patients may not have dispensed some prescriptions or consumed the prescribed medications.

## Conclusion

Our study found a significantly larger number of unique medications were prescribed to patients with public health cover, compared to those without. This difference increased over time and was consistent within all drug classes analysed. As well as reflecting a difference in health cover and access, the groups we examined also relate to differing socioeconomic status. We provide new evidence that the growth in medication burden and polypharmacy among older adults is accelerated for those with public health cover who are typically lower socioeconomic status, which may be helpful in estimating the potential volume of additional prescribing the state may have to provide if public medications entitlement is extended further in Ireland in the future. Such an expansion would provide a further opportunity to assess the impact of extended entitlement on prescribing and medications use.

### Electronic supplementary material

Below is the link to the electronic supplementary material.

Supplementary Material 1

## Data Availability

The data analysed during the current study are not publicly available, as provisions for data sharing were not included in the initial ethical approval, but are available from the corresponding author on reasonable request.
