# Supplementary material for: Prescribing differences among older adults with differing health cover and socioeconomic status: a cohort study
Source: BMC Geriatr. 2023 Nov 17;23:755. doi: 10.1186/s12877-023-04441-9 (PMC10656928; doi:10.1186/s12877-023-04441-9)
Supplement: Supplementary file 1 — Supplementary Material 1 [file 12877_2023_4441_MOESM1_ESM.docx]

# Prescribing differences among older adults with differing health cover and socioeconomic status: a cohort study

## Additional materials


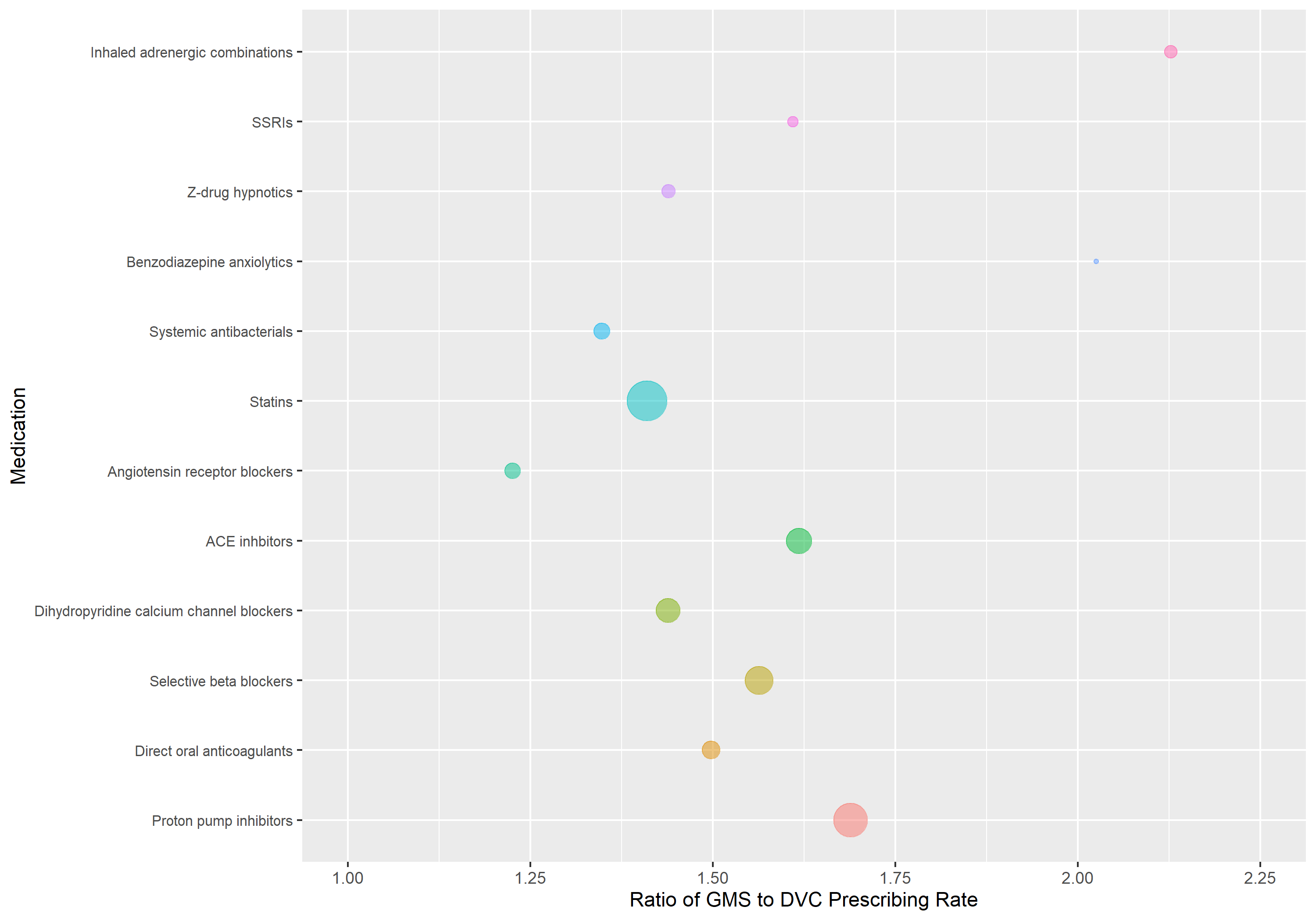

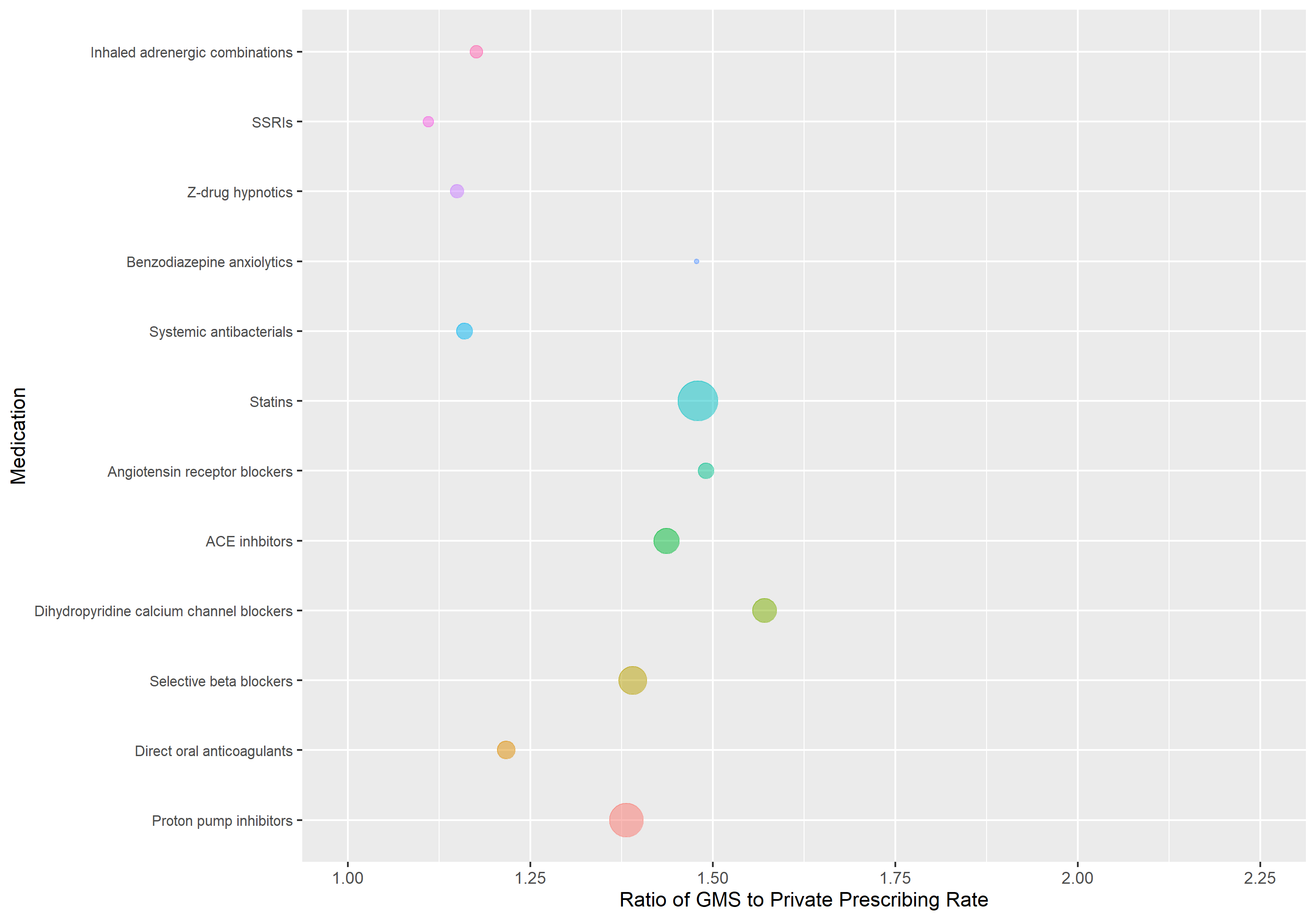


Supplementary figure 1. Ratio of GMS to DVC (top) and GMS to private (bottom) prescribing rates for pre-specified medication classes, with bubble size indicating the rate of prescribing of each class among GMS patients


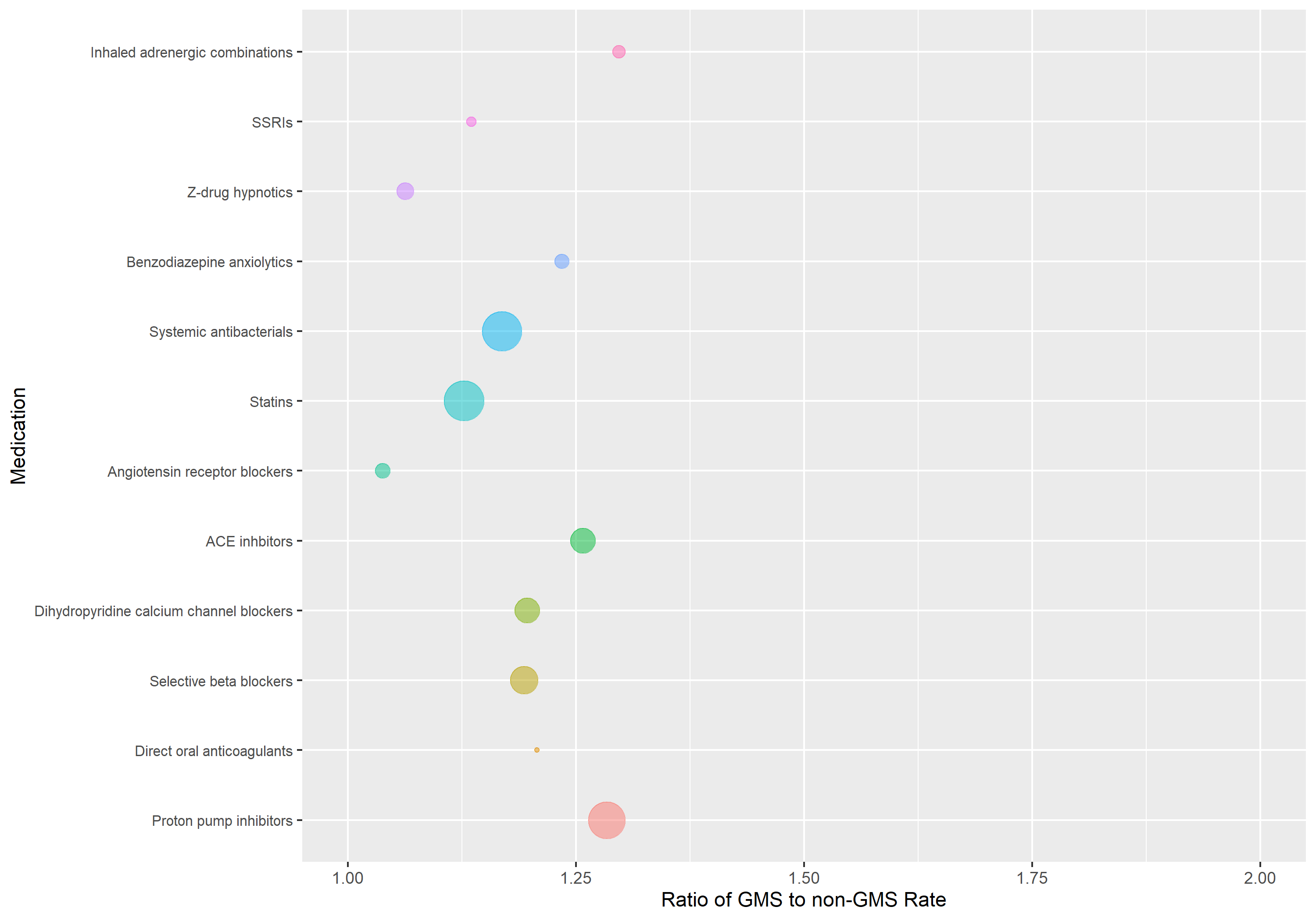


Supplementary figure 2. Ratio of GMS to non-GMS prevalence of prescribing (i.e. percentage of individuals with at least one prescription) for pre-specified medication classes, with bubble size indicating the prevalence of each class among GMS patients


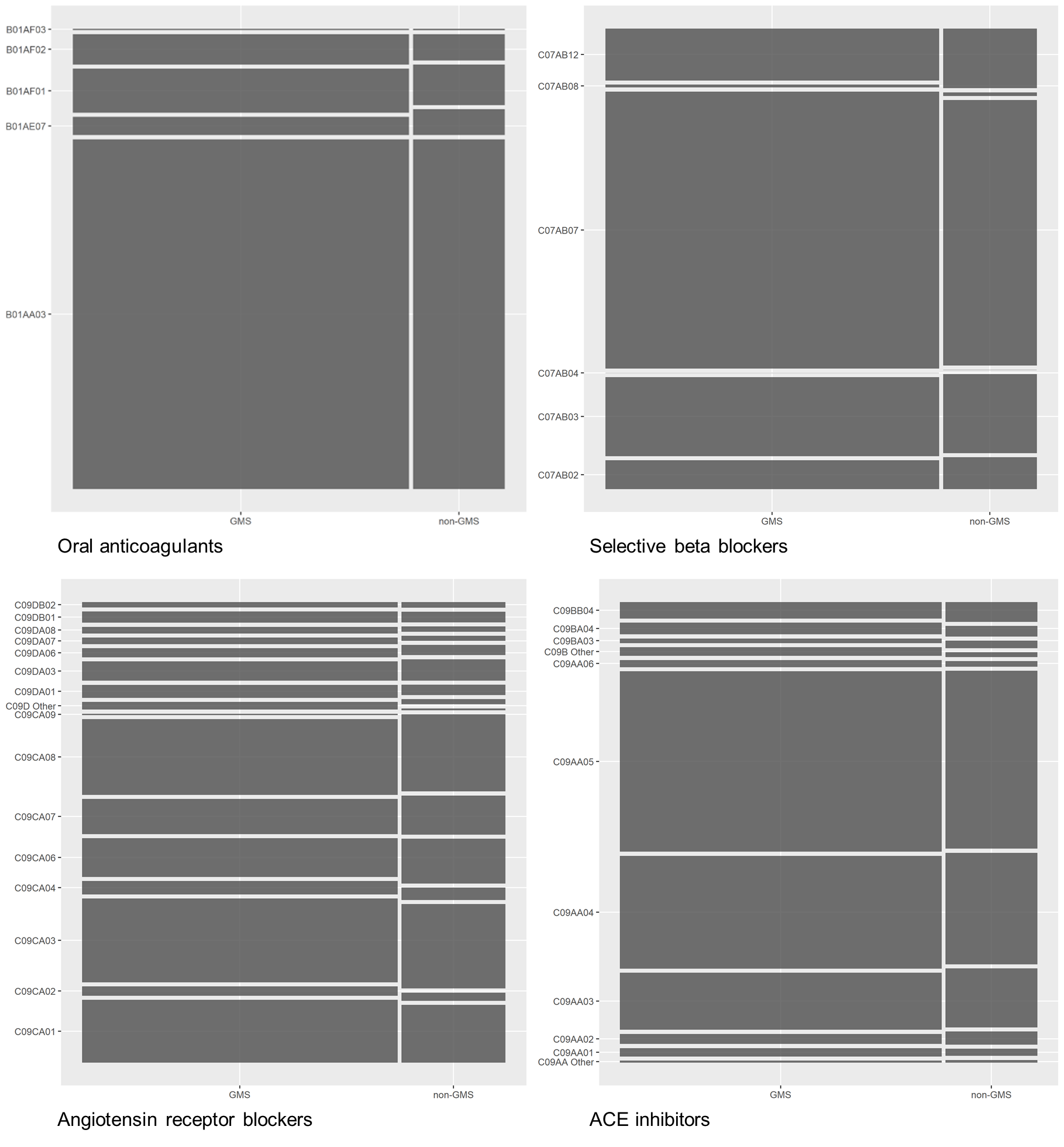


Supplementary figure 3. Relative proportions of individual medication prescribing (indicated by ATC7 codes) for cardiovascular drug classes in GMS and non-GMS groups (clockwise from top left oral anticoagulants, selective beta blockers, ACE inhibitors, and angiotensin receptor blockers)


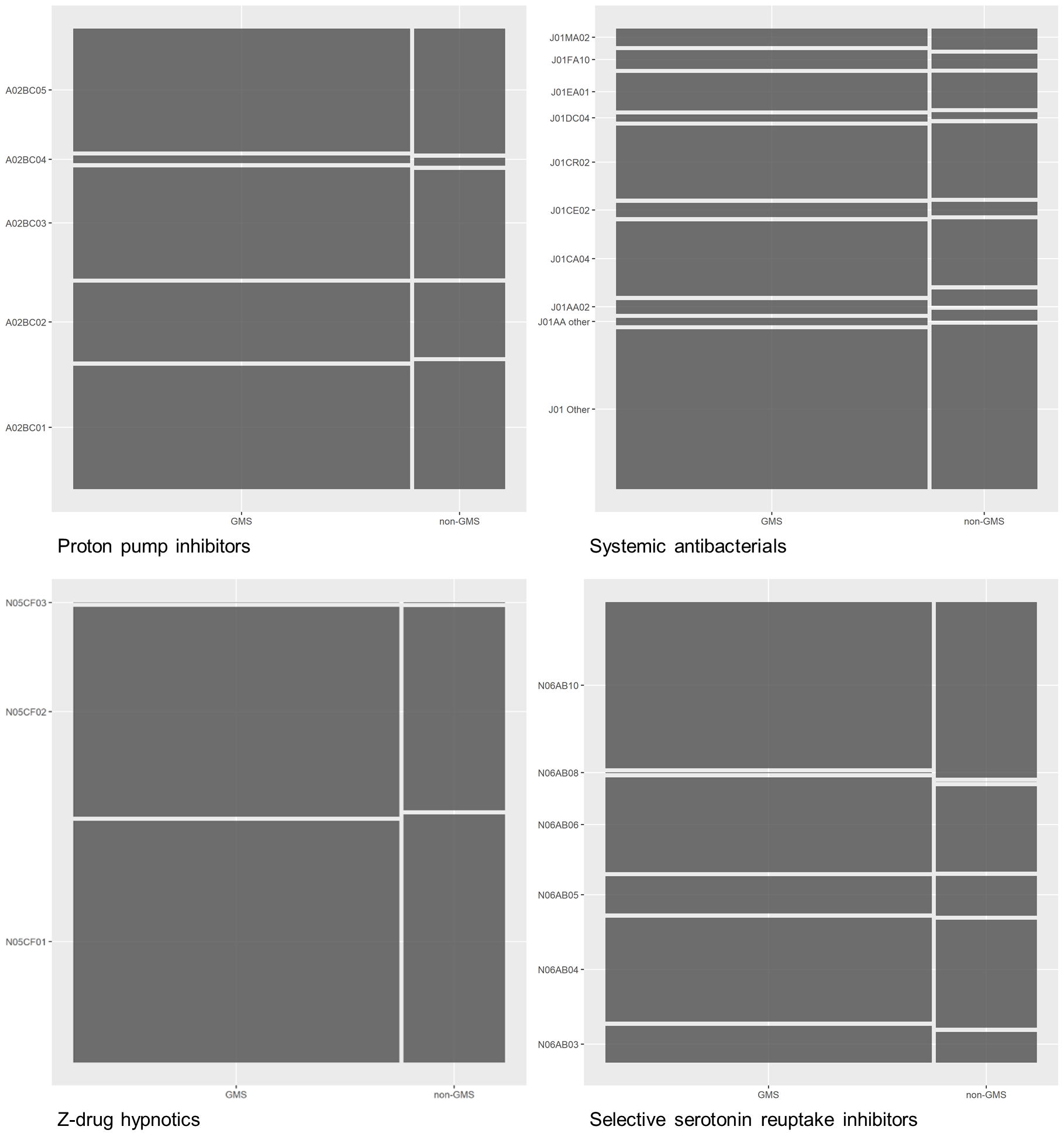


Supplementary figure 4. Relative proportions of individual medication prescribing (indicated by ATC7 codes) for other drug classes in GMS and non-GMS groups (clockwise from top left proton pump inhibitors, systemic antibacterials, selective serotonin reuptake inhibitors and Z-drug hypnotics

Supplementary table 1. Standardised prescribing rates for pre-specified drug classes in GMS and non-GMS groups

|  | **GMS** | | **Non-GMS** | |
| --- | --- | --- | --- | --- |
| **Subgroup** | **Prescriptions** | **Standard rate of prescriptions per 1,000 person years** | **Prescriptions** | **Standard rate of prescriptions per 1,000 person years** |
| PPIs | 586,552 | 4,110 (4,099, 4,120) | 158,142 | 2,630 (2,615, 2,644) |
| ARBs | 204,019 | 1,429 (1,423, 1,436) | 67,089 | 1,057 (1,048, 1,066) |
| SSRIs | 159,771 | 1,119 (1,114, 1,125) | 49,327 | 790 (782, 798) |
| Selective beta blockers | 423,889 | 2,970 (2,961, 2,979) | 118,735 | 1,946 (1,934, 1,958) |
| Systemic antibacterials | 212,795 | 1,491 (1,485, 1,497) | 72,154 | 1,145 (1,136, 1,154) |
| Statins | 787,234 | 5,516 (5,504, 5,528) | 239,687 | 3,759 (3,742, 3,776) |
| ACE inhibitors | 363,001 | 2,543 (2,535, 2,552) | 103,557 | 1,611 (1,600, 1,621) |
| Z-drugs | 181,434 | 1,271 (1,265, 1,277) | 56,383 | 950 (942, 959) |
| Direct oral anticoagulants | 232,610 | 1,630 (1,623, 1,636) | 63,312 | 1,114 (1,105, 1,124) |
| Inhaled adrenergic combinations | 176,463 | 1,236 (1,231, 1,242) | 48,405 | 778 (771, 786) |
| Dihydropyridine CCBs | 341,339 | 2,392 (2,384, 2,400) | 97,381 | 1,568 (1,557, 1,579) |
| Benzodiazepine anxiolytics | 142,097 | 996 (990, 1,001) | 35,295 | 559 (553, 566) |

ACE, angiotensin converting enzyme; ARB, angiotensin receptor blocker; CCB, calcium channel blockers; GMS, General Medical Services; PPI, proton pump inhibitor; SSRI, selective serotonin reuptake inhibitor

Supplementary table 2. Standardised prescribing rates for pre-specified drug classes in GMS, DVC and private groups

|  | **GMS** | | **DVC** | | **Private** | |
| --- | --- | --- | --- | --- | --- | --- |
| **Subgroup** | **Prescriptions** | **Standard rate of prescriptions per 1,000 person years** | **Prescriptions** | **Standard rate of prescriptions per 1,000 person years** | **Prescriptions** | **Standard rate of prescriptions per 1,000 person years** |
| DVC |  |  |  |  |  |  |
| PPIs | 586,552 | 3,801 (3,791, 3,811) | 58,495 | 2,251 (2,230, 2,272) | 99,647 | 2,751 (2,727, 2,775) |
| ARBs | 204,019 | 1,322 (1,316, 1,328) | 30,189 | 1,079 (1,065, 1,093) | 36,900 | 887 (874, 899) |
| SSRIs | 159,771 | 1,035 (1,030, 1,040) | 15,179 | 643 (631, 655) | 34,148 | 933 (919, 947) |
| Selective beta blockers | 423,889 | 2,747 (2,739, 2,755) | 47,608 | 1,757 (1,739, 1,775) | 71,127 | 1,976 (1,956, 1,997) |
| Systemic antibacterials | 212,795 | 1,379 (1,373, 1,385) | 26,046 | 1,023 (1,008, 1,038) | 46,108 | 1,189 (1,173, 1,205) |
| Statins | 787,234 | 5,101 (5,090, 5,113) | 103,155 | 3,618 (3,592, 3,644) | 136,532 | 3,447 (3,421, 3,473) |
| ACE inhibitors | 363,001 | 2,352 (2,345, 2,360) | 40,062 | 1,454 (1,437, 1,471) | 63,495 | 1,638 (1,620, 1,656) |
| Z-drugs | 181,434 | 1,176 (1,170, 1,181) | 20,107 | 817 (804, 830) | 36,276 | 1,023 (1,008, 1,037) |
| Direct oral anticoagulants | 232,610 | 1,507 (1,501, 1,513) | 25,445 | 1,007 (992, 1,021) | 37,867 | 1,238 (1,222, 1,255) |
| Inhaled adrenergic combinations | 176,463 | 1,143 (1,138, 1,149) | 13,943 | 537 (527, 548) | 34,462 | 972 (958, 986) |
| Dihydropyridine CCBs | 341,339 | 2,212 (2,204, 2,219) | 42,557 | 1,537 (1,520, 1,554) | 54,824 | 1,408 (1,392, 1,424) |
| Benzodiazepine anxiolytics | 142,097 | 921 (916, 926) | 11,672 | 455 (445, 464) | 23,623 | 623 (612, 634) |

Supplementary table 3. Number and relative proportions of individual medication prescribing (indicated by ATC7 codes) within pre-specified drug classes for GMS and non-GMS groups

|  |  | **GMS** | | **Non-GMS** | |
| --- | --- | --- | --- | --- | --- |
| **ATC code** | **Drug** | **Prescriptions** | **%** | **Prescriptions** | **%** |
| **PPIs** |  | **586,552** |  | **158,142** |  |
| A02BC01 | Omeprazole | 163,268 | 27.8% | 45,619 | 28.8% |
| A02BC02 | Pantoprazole | 104,198 | 17.8% | 26,518 | 16.8% |
| A02BC03* | Lansoprazole | 146,986 | 25.1% | 38,699 | 24.5% |
| A02BC04 | Rabeprazole | 9,679 | 1.7% | 2,770 | 1.8% |
| A02BC05 | Esomeprazole | 162,421 | 27.7% | 44,536 | 28.2% |
| **Selective beta blockers** |  | **423,889** |  | **118,735** |  |
| C07AB02 | Metoprolol | 27,786 | 6.6% | 8,578 | 7.2% |
| C07AB03 | Atenolol | 75,964 | 17.9% | 21,336 | 18.0% |
| C07AB04 | Acebutolol | 84 | 0.0% | - | 0.0% |
| C07AB07* | Bisoprolol | 267,671 | 63.1% | 71,838 | 60.5% |
| C07AB08 | Celiprolol | 2,496 | 0.6% | 919 | 0.8% |
| C07AB12 | Nebivolol | 49,888 | 11.8% | 16,064 | 13.5% |
| **ACE inhibitors** |  | **363,001** |  | **103,557** |  |
| C09AA01 | Captopril | 7,045 | 1.9% | 1,721 | 1.7% |
| C09AA02 | Enalapril | 8,534 | 2.4% | 3,187 | 3.1% |
| C09AA03 | Lisinopril | 49,454 | 13.6% | 14,571 | 14.1% |
| C09AA04 | Perindopril | 98,127 | 27.0% | 27,679 | 26.7% |
| C09AA05* | Ramipril | 157,049 | 43.3% | 44,171 | 42.7% |
| C09AA06 | Quinapril | 6,047 | 1.7% | 1,328 | 1.3% |
| Other C09AA | Other single ACE inhibitors | 1,619 | 0.4% | 610 | 0.6% |
| C09BA03 | Lisinopril and diuretics | 3,800 | 1.0% | 1,822 | 1.8% |
| C09BA04 | Perindopril and diuretics | 9,995 | 2.8% | 2,577 | 2.5% |
| C09BB04 | Perindopril and amlodipine | 14,028 | 3.9% | 4,808 | 4.6% |
| Other C09B | Other ACE inhibitor combinations | 7,303 | 2.0% | 1,083 | 1.0% |
| **Dihydropyridine CCBs** |  | **341,339** |  | **97,381** |  |
| C08CA01* | Amlodipine | 225,044 | 65.9% | 68,004 | 69.8% |
| C08CA02 | Felodipine | 10,192 | 3.0% | 2,196 | 2.3% |
| C08CA05 | Nifedipine | 10,018 | 2.9% | 3,015 | 3.1% |
| C08CA06 | Nimodipine | 2 | 0.0% | - | 0.0% |
| C08CA10 | Nilvadipine | 1,015 | 0.3% | 124 | 0.1% |
| C08CA13 | Lercanidipine | 95,068 | 27.9% | 24,042 | 24.7% |
| **Z-drugs** |  | **181,434** |  | **56,383** |  |
| N05CF01 | Zopiclone | 97,139 | 53.5% | 30,982 | 54.9% |
| N05CF02 | Zolpidem | 84,229 | 46.4% | 25,347 | 45.0% |
| N05CF03 | Zaleplon | 66 | 0.0% | 54 | 0.1% |
| **SSRIs** |  | **159,771** |  | **49,327** |  |
| N06AB03 | Fluoxetine | 13,366 | 8.4% | 3,435 | 7.0% |
| N06AB04* | Citalopram | 37,806 | 23.7% | 12,122 | 24.6% |
| N06AB05 | Paroxetine | 13,510 | 8.5% | 4,454 | 9.0% |
| N06AB06 | Sertraline | 34,433 | 21.6% | 9,594 | 19.4% |
| N06AB08 | Fluvoxamine | 187 | 0.1% | - | 0.0% |
| N06AB10 | Escitalopram | 60,469 | 37.8% | 19,722 | 40.0% |
| **Benzodiazepine anxiolytics** |  | **142,097** |  | **35,295** |  |
| N05BA01 | Diazepam | 57,856 | 40.7% | 12,829 | 36.3% |
| N05BA02 | Chlordiazepoxide | 2,464 | 1.7% | 918 | 2.6% |
| N05BA04 | Oxazepam | 3 | 0.0% | - | 0.0% |
| N05BA06 | Lorazepam | 7,156 | 5.0% | 1,726 | 4.9% |
| N05BA08 | Bromazepam | 16,862 | 11.9% | 4,880 | 13.8% |
| N05BA09 | Clobazam | 1,431 | 1.0% | 404 | 1.1% |
| N05BA11 | Prazepam | 4,689 | 3.3% | 980 | 2.8% |
| N05BA12 | Alprazolam | 51,636 | 36.3% | 13,558 | 38.4% |
| **ARBs** |  | **204,019** |  | **67,089** |  |
| C09CA01 | Losartan | 32,418 | 15.9% | 9,790 | 14.6% |
| C09CA02 | Eprosartan | 4,612 | 2.3% | 1,344 | 2.0% |
| C09CA03 | Valsartan | 43,252 | 21.2% | 14,309 | 21.3% |
| C09CA04 | Irbesartan | 6,698 | 3.3% | 2,058 | 3.1% |
| C09CA06* | Candesartan | 19,873 | 9.7% | 7,556 | 11.3% |
| C09CA07 | Telmisartan | 18,052 | 8.8% | 6,609 | 9.9% |
| C09CA08 | Olmesartan | 39,000 | 19.1% | 13,055 | 19.5% |
| C09CA09 | Azilsartan | 558 | 0.3% | 270 | 0.4% |
| C09DA01 | Losartan and diuretics | 6,541 | 3.2% | 1,698 | 2.5% |
| C09DA03 | Valsartan and diuretics | 9,841 | 4.8% | 3,594 | 5.4% |
| C09DA06 | Candesartan and diuretics | 4,535 | 2.2% | 1,690 | 2.5% |
| C09DA07 | Telmisartan and diuretics | 3,241 | 1.6% | 805 | 1.2% |
| C09DA08 | Olmesartan and diuretics | 3,304 | 1.6% | 852 | 1.3% |
| C09DB01 | Valsartan and amlodipine | 5,694 | 2.8% | 1,736 | 2.6% |
| C09DB02 | Olmesartan and amlodipine | 2,551 | 1.3% | 896 | 1.3% |
| C09D Other | Other ARB combinations | 3,849 | 1.9% | 827 | 1.2% |
| **Inhaled adrenergic combinations** |  | **176,463** |  | **48,405** |  |
| R03AK06 | salmeterol and fluticasone | 104,141 | 59.0% | 26,391 | 54.5% |
| R03AK07 | formoterol and budesonide | 41,661 | 23.6% | 13,699 | 28.3% |
| R03AK Other | Other adrenergic, corticosteroid combinations | 959 | 0.5% | 176 | 0.4% |
| R03AL02 | Salbutamol and ipratropium bromide | 27,088 | 15.4% | 7,743 | 16.0% |
| R03AL04 | indacaterol and glycopyrronium bromide | 1,984 | 1.1% | 323 | 0.7% |
| R03AL Other | Other adrenergic/ anticholinergic combinations | 630 | 0.4% | 73 | 0.2% |
| **Direct oral anticoagulants** |  | **232,610** |  | **63,312** |  |
| B01AA03 | Warfarin | 183,731 | 79.0% | 50,006 | 79.0% |
| B01AE07 | Dabigatran | 9,446 | 4.1% | 3,675 | 5.8% |
| B01AF01 | Rivaroxaban | 22,991 | 9.9% | 5,763 | 9.1% |
| B01AF02* | Apixaban | 15,880 | 6.8% | 3,697 | 5.8% |
| B01AF03 | Edoxaban | 562 | 0.2% | 171 | 0.3% |
| **Systemic antibacterials** |  | **212,795** |  | **72,154** |  |
| J01AA02 | Doxycycline | 6,827 | 3.2% | 2,752 | 3.8% |
| J01AA other | Other tetracyclines | 3,534 | 1.7% | 1,799 | 2.5% |
| J01CA04 | Amoxicillin | 37,634 | 17.7% | 11,255 | 15.6% |
| J01CE02 | Phenoxymethylpenicillin | 7,135 | 3.4% | 2,228 | 3.1% |
| J01CR02 | Amoxicillin/clavulanic acid | 36,808 | 17.3% | 12,742 | 17.7% |
| J01DC04 | Cefaclor | 3,419 | 1.6% | 1,129 | 1.6% |
| J01EA01 | Trimethoprim | 18,740 | 8.8% | 6,024 | 8.3% |
| J01FA10 | Azithromycin | 9,321 | 4.4% | 2,523 | 3.5% |
| J01MA02 | Ciprofloxacin | 8,684 | 4.1% | 3,542 | 4.9% |
| J01 Other | Other systemic antibacterials | 80,693 | 37.9% | 28,160 | 39.0% |
| **Statins** |  | **787,234** |  | **239,687** |  |
| C10AA01* | Simvastatin | 60,405 | 7.7% | 17,388 | 7.3% |
| C10AA03 | Pravastatin | 91,655 | 11.6% | 25,373 | 10.6% |
| C10AA04 | Fluvastatin | 6,235 | 0.8% | 1,120 | 0.5% |
| C10AA05 | Atorvastatin | 461,117 | 58.6% | 137,523 | 57.4% |
| C10AA07 | Rosuvastatin | 167,822 | 21.3% | 58,283 | 24.3% |

ACE, angiotensin converting enzyme; ARB, angiotensin receptor blocker; CCB, calcium channel blockers; GMS, General Medical Services; PPI, proton pump inhibitor; SSRI, selective serotonin reuptake inhibitor
